# A huge Omental Lymphangioma with extention into Labia Majorae: A case report

**DOI:** 10.1186/1471-2482-6-18

**Published:** 2006-12-27

**Authors:** Prashant N Mohite, Ashok M Bhatnagar, Shailesh N Parikh

**Affiliations:** 1 Department of Surgery, SSG (Government) Hospital & Medical College, Sayajiganj, Vadodara, Gujarat State, India

## Abstract

**Background:**

Abdominal cystic lymphangiomas are uncommon congenital benign tumors.

**Case presentation:**

We present a case of a 4 year old female child with a cystic lymphangioma arising from greater omentum and occupying whole of the abdomen and protruding through labia mejora. Ultrasonography and CT scan confirmed the diagnosis. Complete excision of the cyst along with omentectomy done with no clinical or radiological evidence of recurrence till 6 months.

**Conclusion:**

Due to variable presentation of abdominal lymphangiomas, extensive imaging studies are necessary for evaluation and diagnosis. Complete surgical resection is a treatment of choice.

## Background

Ninety-five percent of lymphangiomas occur in the head and axila, while intra-abdominal lesions (located in the omentum, mesentery and retroperitoneum) are rare. Lymphangiomas are hamartomas of the lymphatic vessels that have the potential to infiltrate surrounding structures. About 50% are present at birth and up to 90% become evident by the age of two years. The prognosis of lymphangiomas depends on the location and extent of the lesion and the presence of other associated abnormalities. Complete resection is the treatment of choice and has an excellent prognosis. Although an abdominal lymphangioma is considered benign, it may become locally invasive. Therefore any involved organ must also be resected. Incomplete resection may lead to recurrence. Follow-up imaging is advised, with ultrasound as the modality of choice.

## Case presentation

**Figure 1:**
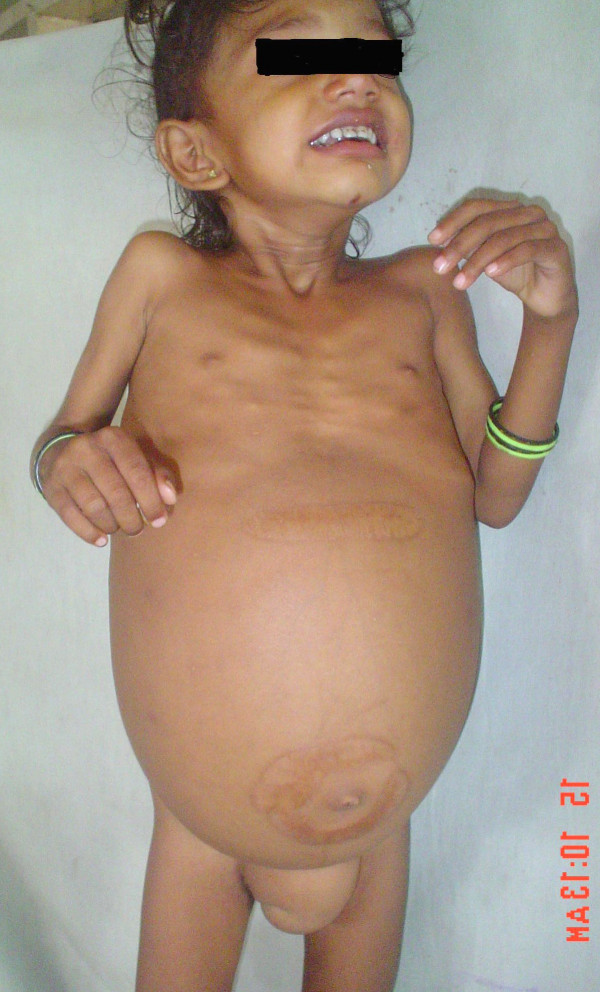
Patient on presentation.

A 4 year old female presented with huge abdominal swelling first noticed by her parents at 2 years of her age (See Figure 1). The swelling had increased in size since then to occupy almost whole of the abdomen. Right labia had a swelling which increases in size on standing and decrease on lying down. On examination abdomen was found to be distended, felt tense with flanks full. The swelling over the labia was reducible on manipulation suggesting its continuity with the abdominal swelling.

**Figure 2:**
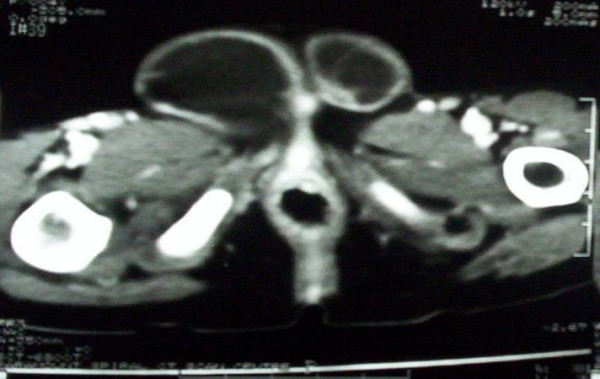
CT scan of abdomen-pelvis showing extension of the cyst into labia mejorae on both sides.

Ultrasound abdomen showed 30 × 20 × 20 centimeters huge, multiloculated, multiseptate, lesion extending from xiphisternum to pelvis with extension into the right labia majora. Lesion was causing lateral and posterior displacement of bowel loops and compression of liver and both kidneys. A provisional diagnosis of omental, mesenteric or ovarian cyst was made. CT scan was done to throw some more light on the site of origin. Non-contrast film showed the cyst filled with clear fluid possibly arising from the mesentery. Contrast film was suggestive of multiloculated cyst. Pelvic films proved the labial extention of the cyst (See Figure 2), but still the exact site of origin remained unclear.

**Figure 3:**
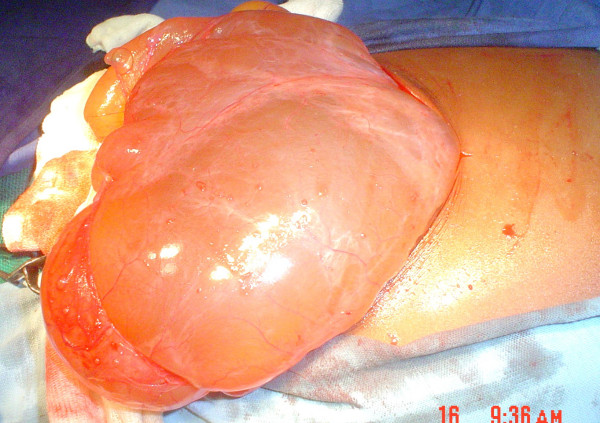
Thin walled cyst popping out of abdominal incision.

**Figure 4:**
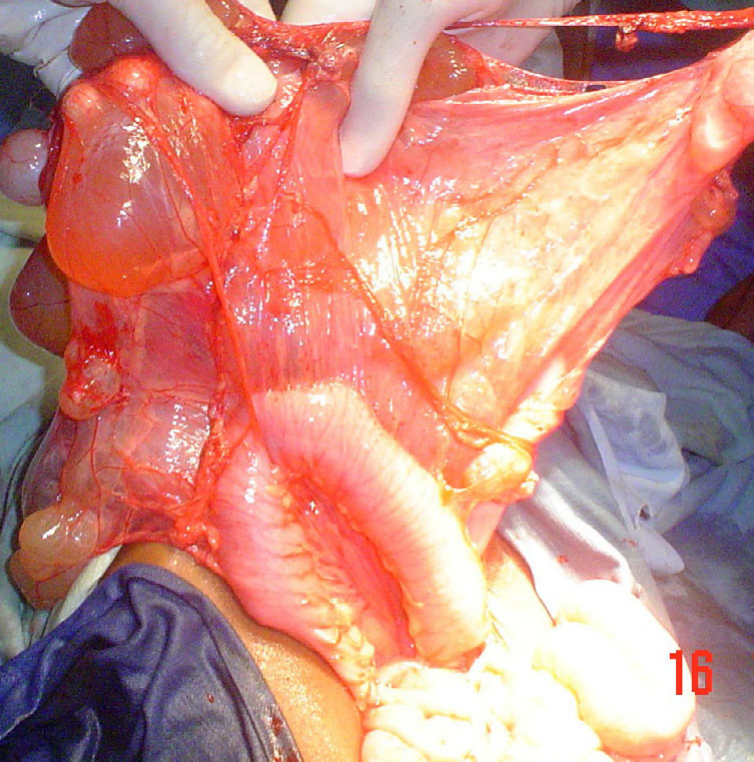
Intraoperative picture showing cyst arising from omentum.

Patient was taken up for exploratory laparotomy and midline vertical incision was kept. After opening the peritoneum a thin walled cyst was seen popping out of the skin incision (See Figure 3). On exploration it was found attached to the greater omentum (See Figure 4).

**Figure 5:**
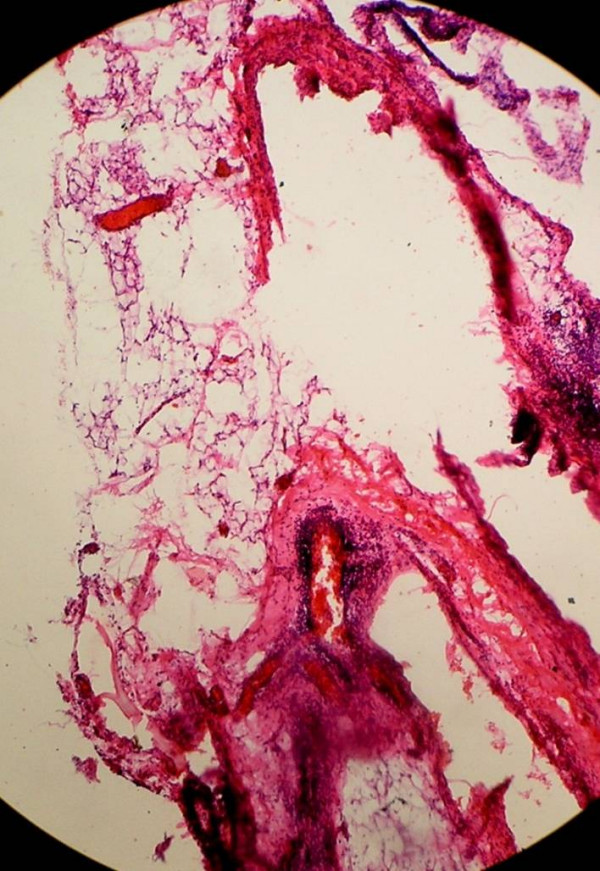
Microscopic view of the cyst wall.

Cyst was deflated partially by aspiration and we found the swelling over labia majora got reduced as we deflated the cyst and no adhesions were found between the cyst wall and labia. Complete excision of the cyst wall along with part of omentum was done. Histopathological report of the cyst wall showed endothelial cell lining, foam cells, lymphatic spaces, lymphoid tissue and smooth muscles which are diagnostic of lymphangiomatous cyst (See Figure 5). Patient was under follow up for since six months and had no clinical and radiological signs of recurrence.

## Discussion

Abdominal cystic lymphangiomas are uncommon congenital benign tumors [1]. The mean age at presentation is 2.2 years. The study performed by Konen et al [2] showed that the male-to-female ratio is 5:2. Etiology [3] may be benign proliferations of ectopic lymphatics that lack communication with the normal lymphatic system. Cysts are thought to arise from lymphatic spaces associated with the embryonic retroperitoneal lymph sac. Failure of the embryonic lymph channels to join the venous system, trauma, neoplasia and degeneration of lymph node are some proposed etiologies.

Clinical presentation [4] may be chronic in which there is gradual distention in abdomen with or with out pain. It may present in acute form in which there is acute pain, distention, fever, vomiting and peritonitis. There may be features of small bowel obstruction which may occur by extrinsic luminal compression, by traction on the mesentery or by volvulus.

A well-circumscribed anechoic mass with posterior acoustic enhancement is a typical ultrasound presentation [5]. CT scan shows well defined, thin walled multiseptate lesion and distinguished from ascites by the absence of bowel loop separation or fluid in the typical sites, such as the cul-de-sac [6]. Complications [7] are intestinal obstruction (most common), volvulus, hemorrhage into the cyst, infection, rupture, cystic torsion and obstruction of the urinary and biliary tract. Complete resection is the treatment [8] of choice and has an excellent prognosis.

Although an abdominal lymphangioma is considered benign, it may become locally invasive. Therefore any involved organ must also be resected. Incomplete resection may lead to recurrence[8]. If the patient was treated with marsupialization, closer follow-up for possible recurrence should be instituted. Otherwise, no long-term follow-up for surgical problems is necessary. The recurrence rate ranges from 0–13.6%. If cysts are discovered prenatally, intervention during early infancy is indicated to prevent potential complications such as obstruction and intestinal volvulus. In our patient the cyst was found in the greater omentum which was excised completely without bowel resection.

## Conclusion

Intraabdominal lymphangiomas are benign lesions but considering their local invasive property complete resection of the cyst along with involved organ and clinical and radiological follow up for recurrence is necessary.

## Declaration of competing interests

The author(s) declare that they have no competing interests.

## Authors' contributions

All authors read and approved the final manuscript.

1. PNM -Helped in radiological investigations

-Helped in histopathological diagnosis

-Assisted the surgery

-Drafted the manuscript

2. AMB -Gave guidance in surgery

-Gave final touch and approval to the manuscript

-Coordination

3. SP -Did the surgery

-Took postoperative care

-Participated in designing and revising the manuscript

## Pre-publication history

The pre-publication history for this paper can be accessed here:
